# Stem cell therapy for cardiac dysfunction

**DOI:** 10.1186/2193-1801-3-440

**Published:** 2014-08-19

**Authors:** Amer A Matar, James JH Chong

**Affiliations:** Sydney Medical School, University of Sydney, Sydney, NSW Australia; Department of Cardiology, Westmead Hospital, Sydney, NSW Australia; Centre for Heart Research, Westmead Millennium Institute, Sydney, NSW Australia

**Keywords:** Stem cells, Myocardial infarction, Cardiac regeneration

## Abstract

Following significant injury, the heart undergoes induced compensation and gradually deteriorates towards impending heart failure. Current therapy slows but does not halt the resultant adverse remodeling. Stem cell therapy, however, has the potential to regenerate or repair infarcted heart tissue and therefore is a promising therapeutic strategy undergoing intensive investigation. Due to the wide range of stem cells investigated, it is difficult to navigate this field. This review aims to summarize the main types of stem cells (both of cardiac and extra-cardiac origin) that possess promising therapeutic potential. Particular focus is placed on clinical trials supporting this therapeutic strategy.

## Introduction

Myocardial infarction (MI) remains a leading cause of death and disability worldwide. In the United States alone, approximately 1 million cases of MI occur annually (Roger et al. 2011). The implementation of cardiovascular prevention strategies continues to reduce the incidence of MI. Concurrently, however, evolution of pharmacological approaches and coronary reperfusion interventions has led to an increased post-MI survival rate, which in turn has raised MI disease morbidity. Previous estimates indicate that approximately 90–95% of patients survive their first MI (Rosamond et al. 2012), contributing to a current “epidemic” of heart failure and imposing an enormous health burden on individuals and the community.

After MI, local cardiac compensatory mechanisms are activated giving rise to a vicious cycle of cardiac metabolic insufficiency, leading to heart failure and potentially sudden death (Orn et al. 2007). Timely reperfusion together with optimal drug and device-based interventions has improved MI management by reducing the initial burden of injury and slowing progression of resultant adverse remodeling (White et al. 2005). Nevertheless, no current therapy is able to reverse the inexorable decline in cardiac function. Therefore, new strategies investigating cardiac regeneration have demanded considerable interest. These involve either 1) the implantation of stem cells or their derivatives directly into the heart or 2) the activation of endogenous cardiac repair mechanisms in order to replace damaged cardiomyocytes and promote vascular reconstruction (Laflamme and Murry 2011).

This review provides physicians with a concise overview of the major types of stem cells, both from cardiac or extra-cardiac origins, being investigated for post-MI treatment. For further detail and information on endogenous cardiac regeneration, the interested reader is directed to the following detailed reviews (Laflamme and Murry 2011; Choi and Poss 2012; Rasmussen et al. 2011).

## Extra-cardiac stem cells

### Skeletal myoblasts

Skeletal myoblasts (SKM) are the progenitor cells of skeletal muscle. Initial observations that SKMs could be harvested from an autologous origin, easily expanded ex vivo and undergo spontaneous differentiation into contractile muscle sparked interest in SKMs for cardiac myoplasty (Taylor et al. 1998). Early uncontrolled clinical studies reported that SKMs could engraft in the injured heart with remarkable efficiency and enable significant improvement in cardiac function (Menasche et al. 2003). These findings were not reproduced in a subsequent prospective randomized placebo-controlled trial (Menasche et al. 2008), where 97 participants with severe left ventricular (LV) dysfunction underwent transepicardial autologous SKM injection at the time of coronary artery bypass grafting. Six months following the procedure, no improvement in LV function was found when compared to placebo. Importantly, a high prevalence of ventricular tachyarrhythmias was observed leading to premature discontinuation of the trial. Similar results were observed in the SEISMIC trial which used transendocardial injection of autologous SKMs (Veltman et al. 2008). A follow-up study conducted four years later reported no significant change in LV function compared to the placebo group.

The general consensus amongst clinicians now is that SKMs do not electrically couple to host cardiomyocytes (Leobon et al. 2003). Notably, it is now understood that the gap-junction protein, connexin 43, can augment intracellular coupling of cardiomyocytes and confers a protective effect against ventricular tachyarrhythmias following cell transplantation (Roell et al. 2007).

### Bone marrow mononuclear cells

The major stem cell type in the bone marrow (BM) is the hematopoietic stem cell (HSC). HSCs comprise less than 0.1% of unfractionated bone marrow mononuclear cell (BMMNC) samples (Challen et al. 2010). Although the vast majority of BMMNCs are not stem cells, they are still considered a significant source of hematopoietic progenitors which may be useful for cardiac repair. In 2001, a landmark murine study administered BM- derived HSCs by direct intramyocardial injection into infarcted murine hearts (Orlic et al. 2001). The transplanted cells reportedly underwent transdifferentiation directly into cardiomyocytes and supporting vasculature leading to improved LV function. These results were wholeheartedly embraced by clinicians and a wave of clinical trials using BMMNCs for cardiac regeneration ensued. The first of these reports using BM cardiac cell therapy appeared only months after this initial murine publication (Strauer et al. 2001) which proved to be highly controversial. Several high profile groups have been unable to replicate its findings (Murry et al. 2004; Balsam et al. 2004). It is now generally believed that transdifferentiation of HSCs to cardiomyocytes does not occur to any meaningful degree. Importantly however, cardiac cell therapy with BMMNCs may rely on other mechanisms to achieve favorable cardiac repair after MI. This may include secretion of growth factors and other proteins capable of promoting angiogenesis (Ruger et al. 2008) and endogenous cardiac stem cell or cardiomyocyte proliferation (Laflamme and Murry 2011).

Clinical trials using BMMNCs for cardiac repair have now been the subject of several meta-analyses (Abdel-Latif et al. 2007; Martin-Rendon et al. 2008; Clifford et al. 2012). Together, close to 2000 subjects have received BMMNCs as cell therapy for cardiac dysfunction (predominantly for ischemic cardiomyopathy). The pooled results suggest that this treatment appears safe but clinical improvements are modest. In fact, the very recent phase 2 FOCUS-CCTRN trial reported no significant improvement in LV end-systolic volume, further undermining the efficacy of BMMNCs (Perin et al. 2012). Notably, most of the earlier trials used endpoints based on LV functional imaging or subjective symptom based questionnaires. No study has definitively examined hard clinical endpoints such as mortality. To this end, a large randomized multi-center European clinical trial investigating intracoronary delivery of BMMNCs after MI has commenced enrolment with its primary endpoint being all-cause mortality (NCT01569178) (Table 1). If results are positive, this may lead to increased uptake of this novel treatment by clinical cardiologists.

**Table 1:** **Recent and ongoing clinical trials involving extra-cardiac stem cells in patients with ischemic cardiomyopathy**

Trial name/Investigator	Study identifier	Comparators	Endpoint	Patients	Delivery route	Type classification
**SKMs**						
MARVEL	NCT00526253	Low dose vs high dose vs placebo	Safety + QOL	170	Intramyocardial	Safety + efficacy, Phase 1/2
**BMCs**						
REPAIR-AMI*	NCT00279175	BMC vs placebo	LVEF	204	Intracoronary	Efficacy, Phase 3
REGEN-IHD	NCT00747708	Intracoronary BMC + G-CSF vs intramyocardial BMC + G-CSF vs G-CSF vs placebo	LVEF	148	Intracoronary/Intramyocardial	Safety + efficacy, Phase 2/3
BAMI	NCT01569178	BMC vs no intervention	All-cause mortality	3000	Intramyocardial	Safety + efficacy, Phase 3
REPEAT	NCT01693042	Single vs repeated (2 times) BMC infusions	Mortality + morbidity	676	Intracoronary	Safety + efficacy, Phase 2/3
FOCUS*	NCT00824005	BMMNC vs placebo	LVESV	92	Intramyocardial	Safety + efficacy, Phase 2
ASSURANCE	NCT00869024	BMMNCs infusion	Mortality + morbidity	24	Intramyocardial	Safety, Phase 1/2
REVITALIZE	NCT00874354	BMMNCs infusion	LVEF	30	Intracoronary	Safety + efficacy, Phase 1
**EPCs/CD133+ Cells**						
PERFECT*	NCT00950274	CD133+ vs placebo	LVEF	142	Intramyocardial	Efficacy, Phase 3
Cardio133*	NCT00462774	CD133+ vs placebo	LVEF	60	Intramyocardial	Efficacy, Phase 2/3
IMPACT-CABG	NCT01033617	CD133+ vs placebo	SAE	20	Intramyocardial	Safety + efficacy, Phase 2
AlsterMACS	NCT01337011	Intracoronary vs intramyocardial CD133+ infusions	LVEF	64	Intracoronary/Intramyocardial	Efficacy, Phase 1/2
SELECT-AMI	NCT00529932	CD133+ vs placebo	LV wall thickness	60	Intracoronary	Safety + efficacy
**EPCs/CD133+ Cells vs BMCs**						
Baharvand et al.	NCT01167751	BMMNC vs CD133+ vs placebo infusions	LVEF	105	Intracoronary	Safety + efficacy, Phase 3
Ghassemi et al.	NCT01187654	BMMNC vs CD133+ vs placebo infusions	LVEF	80	Intracoronary	Safety + efficacy, Phase 2/3
**MSCs**						
Adipose Tissue MSCs						
ATHENA	NCT01556022	MSCs vs placebo	SAE + LVEF	45	Intramyocardial	Safety + efficacy, Phase 2
ADVANCE	NCT01216995	MSCs vs placebo	SAE + Infarct size	216	Intracoronary	Safety + efficacy, Phase 2
Parcero et al.	NCT01502514	MSCs infusion	QOL	10	Intramyocardial	Safety + efficacy, Phase 1/2
Umbilical Cord MSCs						
Yan et al.	NCT01946048	MSCs vs placebo	LVEF	10	Intramyocardial	Safety + efficacy, Phase 1
Bone Marrow MSCs						
ESTIMATION	NCT01394432	MSCs vs placebo	LVESV	50	Intramyocardial	Efficacy, Phase 3
SEED-MSC	NCT01392105	MSCs vs no intervention	LVEF	80	Intracoronary	Safety + efficacy, Phase 2/3
Anastasiadis et al.	NCT01753440	Allogeneic MSCs	LVEF	30	Intramyocardial	Safety + efficacy, Phase 2/3
Anastasiadis et al.	NCT01759212	Allogeneic MSCs	LVF	10	Intramyocardial	Safety + efficacy, Phase 2/3
Perin et al.	NCT00555828	25 vs 75 vs 150 million allogeneic MSCs vs placebo	Safety + LVF	25	Intramyocardial	Safety + efficacy, Phase 1/2
PROMETHEUS*	NCT00587990	Low vs high dose MSCs vs placebo	SAE	45	Intramyocardial	Safety + efficacy, Phase 1/2
MESAMI	NCT01076920	MSCs infusion	Safety + LVF	10	Intramyocardial	Safety, Phase 1/2
MSC-HF	NCT00644410	MSCs vs placebo	LVF	60	Intramyocardial	Safety + efficacy, Phase 1/2
Allogeneic vs Autologous MSCs						
POSEIDON-Pilot*	NCT01087996	Auto-MSCs (20, 100 or 200 million) vs Allo-MSCs (20, 100 or 200 million)	SAE + LVF	30	Intramyocardial	Safety + efficacy, Phase 1/2
BMCs vs MSCs						
TAC-HFT*	NCT00768066	MSCs (100 or 200 million) vs BMCs (100 or 200 million) vs placebo	SAE + LVF	67	Intramyocardial	Safety + efficacy, Phase 1/2

All trials use autologous infusions unless otherwise stated. BMC-Bone Marrow Stem Cell, BMMNC-Bone Marrow Mononuclear Cell, EPC-Endothelial Progenitor Cell, G-CSF-Granulocyte-Colony Stimulating Factor, LVEF-Left Ventricular Ejection Fraction, LVESV-Left Ventricular End Systolic Volume, LVF-Left Ventricular Function, MSC-Mesenchymal Stem Cell, QOL-Quality of Life, SAE-Serious Adverse Events, SKM-Skeletal Myoblast.

*Denotes trials with published results (including preliminary results).

### Mesenchymal stem cells

In addition to HSCs, mesenchymal stem cells (MSCs) represent another group of stem cells found in the BM, as well as other tissues such as adipose tissue and cord blood. MSCs (also known as mesenchymal stromal cells or colony-forming unit fibroblasts) were first isolated over 40 years ago (Friedenstein et al. 1970) and have been shown to directly transdifferentiate into cardiomyocytes in the presence of the demethylating agent 5-azacytidine (5-AZA) (Wakitani et al. 1995) or when co-cultured with other cardiomyocytes (Rangappa et al. 2003; Li et al. 2007). Furthermore, MSCs possess several inherent features which facilitate their use in a clinical setting. In addition to being easily harvested and cultured ex vivo, MSCs are believed to be able to modulate the host immune system via lymphocyte regulation (Di Nicola et al. 2002) and the suppression of inflammatory cytokine release from cells of the innate immune system (Aggarwal and Pittenger 2005). These characteristics render MSCs a promising allogeneic cell source for treatment of infarcted hearts.

As with BMMNCs however, controversy surrounds their cardiomyogenic potential. In a study by Rose et al., BM-derived MSCs (BM-MSCs) did not transdifferentiate into functional cardiomyocytes in vitro (Rose et al. 2008) but were able to express cardiomyocyte specific proteins. Regardless, MSCs remain the subject of considerable interest for cardiac repair strategies. They rapidly progressed through the pre-clinical arena, where small and large animal studies suggested potential to improve cardiac function after induced MI (Shake et al. 2002; Schuleri et al. 2009). Eventually clinical trials ensued. The first of these was a randomized, double blinded, placebo controlled phase 1 dose escalation study using intravenous allogeneic BM-MSC infusions after MI (Hare et al. 2009). Here, MSC treatment appeared safe after twelve months of follow up. Unexpectedly, when addressing the arrhythmogenic potential of MSC transfusions, ambulatory electrocardiogram monitoring showed that arrhythmic risk had decreased. The investigators of this trial reported an improvement in the global symptom score at 6 months and a significant improvement in LV function at 3 but not 6 months. The latter finding was attributed to a “catch-up” phenomenon in ventricular function of placebo treated patients. Interestingly, similar “catch-up” has been reported in BMMNC therapy trials (Meyer et al. 2006).

Recent trials also appear to support MSC derived improvements on cardiac dysfunction Table 1. The phase 1/2 randomized POSEIDON trial investigated autologous compared to allogeneic MSCs in patients undergoing coronary artery bypass grafting for ischemic cardiomyopathy. Initial published results documented safety and overall efficacy (Hare et al. 2012). Interestingly, further analysis of imaging data found that the functional effects of MSCs appeared preferentially at local sites of MSC injection whilst scar reduction was seen more globally (Suncion et al. 2014). Similar effects of MSCs on scar were seen by Williams et al. who used percutaneous transendocardial injections (Williams et al. 2011). The Transendocardial Autologous Cells in Ischemic Heart Failure Trial (TAC-HFT) also used percutaneous intramycoardial injections to compare efficacy of MSCs to BMMNCs (Heldman et al. 2014). MSCs, but not BMMNCs, reduced infarct size and improved regional myocardial function. The Prospective Randomized Study of MSC Therapy in Patients Undergoing Cardiac Surgery (PROMETHEUS) study used magnetic resonance imaging to investigate mechanisms by which MSCs may improve LV function (albeit in a very limited sample population of six patients) (Karantalis et al. 2014). Myocardial segments injected with MSCs showed not only a reduction in scar size with corresponding increased contractile improvement, but also increased perfusion despite the lack of coronary artery bypass to these segments.

A different and novel approach was used in the recent C-CURE trial. Here the therapeutic effect of BM-MSCs exposed to a cytokine cocktail designed to induce partial cardiogenic differentiation was investigated (resulting cells were named cardiopoietic stem cells) (Bartunek et al. 2013). The autologous cardiopoietic stem cells were delivered via transendocardial injection into the LV myocardium of patients suffering from an ischemic cardiomyopathy. Results showed that this strategy was feasible and appeared safe. Furthermore, LV function was significantly improved in the cardiopoietic stem cell therapy group compared to placebo (Bartunek et al. 2013).

**Figure 1:**
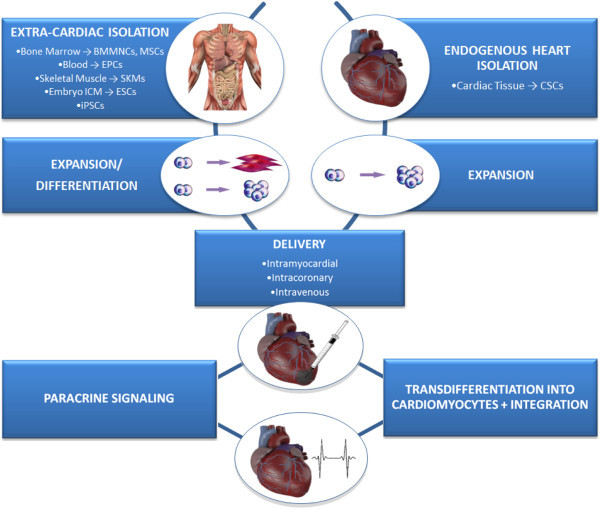
**Schematic representation of the potential sequence of events involved in successful regenerative stem cell treatment of cardiac tissue in an infarcted heart.** BMMNC ‒ Bone Marrow Mononuclear Cell, CSC ‒ Cardiac Stem Cell, EPC ‒ Endothelial Progenitor Cell, ESC ‒ Embryonic Stem Cell, ICM ‒ Inner Cell Mass, iPSC ‒ Induced Pluripotent Stem Cell, MSC ‒ Mesenchymal Stem Cell, SKM ‒ Skeletal Myoblast.

It is important to note that the mechanisms underpinning effects of MSC cardiac therapy are not yet completely understood. Inconsistent pre-clinical results demonstrating MSC differentiation into cardiomyocytes may be attributed to varied methods of MSC isolation and propagation. Significant MSC to cardiomyocyte transdifferentiation in the clinical trials discussed above seems unlikely since MSCs engraft poorly in cardiac tissue. Theories have now shifted to support a more indirect mechanism involving paracrine mediators that in turn contribute to angiogenesis or new host cardiomyocyte formation (either from resident cardiac stem cells or division of existing cardiomyocytes) (Williams et al. 2011, 2013; Li et al. 2010) (Figure 1).

Regarding origins, MSCs have reportedly been isolated from virtually all post-natal organs (da Silva Meirelles et al. 2006), including the heart (Chong et al. 2011, 2013). In particular, adipose tissue derived MSCs (AD-MSCs) have become the subject of recent research efforts. In preclinical rodent and porcine studies, these MSCs were shown to induce angiogenesis and significantly improve LV function (Valina et al. 2007; Cai et al. 2009). As a result, several clinical trials using AD-MSCs are currently being conducted (Table 1).

### Endothelial progenitor cells

Early reports of another stem/progenitor cell population surfaced in 1997, when Asahara et al. described a population of BM-derived cells expressing CD34 (HSC marker) (Asahara et al. 1997). Notably, CD34 is also expressed in a subset of endothelial cells. These cells were distinct from other BM-derived stem cell populations and were named endothelial progenitor cells (EPCs) due to their ability to differentiate ex vivo towards an endothelial cell lineage. Although expressing the cell surface proteins VEGFR2 and CD133 (Asahara et al. 1997; Yin et al. 1997), these markers are ubiquitously expressed, and hence EPCs lack a definitive marker for prospective identification and require in vitro assays for isolation and propagation.

The cardiac repair ability of EPCs lies in their potential to promote neovascularization, possibly by both direct (by differentiation into endothelial cells) and indirect (mediated by angiogenic growth factors) mechanisms. They are implicated in wound healing throughout the body. For this reason, these cells continue to be studied and may contribute clinically to cardiac repair post-MI as well as to angiogenesis in patients with refractory angina (Friis et al. 2011). This is the focus of a currently recruiting phase 3 clinical trial called Efficacy and Safety of Targeted Intramyocardial Delivery of Auto CD34+ Stem Cells for Improving Exercise Capacity in Subjects With Refractory Angina (RENEW) (NCT01508910).

### Pluripotent stem cells (embryonic stem cells/induced pluripotent stem cells)

Embryonic stem cells (ESCs) extracted from the inner cell mass of a blastocyst (early-stage embryo) are able to give rise to cells of any of the 3 germ layers (Eckfeldt et al. 2005). In the correct culture conditions, ESCs can differentiate into many different cells from varied organs. These cells thus possess formidable therapeutic potential and have been extensively studied. Three major concerns, however, have slowed their clinical translation. Firstly, undifferentiated pluripotent stem cells harbor great tumorigenic potential. Inadvertent transplantation of these undifferentiated cells poses significant risks to the receiving host. Secondly, ESCs will necessarily be used as an allogeneic product which is likely to induce host immune rejection following transplantation. Finally, ethical concerns held by some groups have created political hurdles in several countries. To some degree, the limitations above have been addressed and clinical trials using ESC-derived therapy have now become a reality (Schwartz et al. 2012) (also see the ongoing ‘Safety Study of GRNOPC1 in Spinal Cord Injury’ trial, NCT01217008). With regards to cardiac therapy, several pre-clinical studies have demonstrated the ability of mouse (Min et al. 2002) and human (Laflamme et al. 2007; Fernandes et al. 2010; Pearl et al. 2011; Chong et al. 2014) ESC-derived cardiomyocytes to engraft and repair the infarcted heart.

In 2006, a group led by Shinya Yamanaka reported the possibility of reprogramming differentiated somatic cells into a pluripotent state similar to ESCs (Takahashi and Yamanaka 2006). These were named induced pluripotent stem cells (iPSCs) and the initial murine studies were replicated with human cells (Takahashi et al. 2007). This method was embraced by scientists as a means to overcome the ethical dilemmas associated with ESC use. Furthermore, iPSCs could provide a theoretical means for a myriad of cell types to be made from a recipient’s own somatic cells. The resultant cell therapy products would be syngenic (genetically identical) and theoretically would circumvent the host’s immune system. Nevertheless, T-cell mediated immune rejection was evident in murine studies despite the use of syngenic iPSCs (Zhao et al. 2011). In summary, pluripotent stem cells bare undeniable potential for large scale cardiac regeneration, but additional concerns must be addressed systematically to utilize their vast therapeutic abilities. The interested reader is directed to the following review covering recent translational efforts in this area (Chong and Murry 2014).

## Endogenous cardiac stem cells

The notion of the heart being a terminally differentiated organ was first challenged almost a decade ago. Here, investigators reported the presence of possible endogenous cardiac stem cells (CSCs) in the human adult heart (Nadal-Ginard et al. 2003). In human sex-mismatched cardiac transplants, female hearts transplanted into male hosts had a significant number of chromosome Y positive cardiomyocytes and coronary vessels expressing the stem cell markers c-kit, mdr1 or Sca-1 (Quaini et al. 2002), suggesting that the adult heart may not be quiescent as previously thought. Since then, several cardiac stem cell populations have been isolated from various species, including humans. These populations possess the cardinal characteristics of stem cells, namely long term self-renewal, clonogenicity and multipotency, hence their regenerative potential holds great promise for cardiac therapeutics. In addition, it is expected that the restrictions in cardiomyocyte differentiation of extra-cardiac adult stem cells arises from epigenetic phenotypic restriction imposed on cells not of cardiac origin. To this end, it is likely that CSCs have greater potential for differentiation to cardiac cells, including cardiomyocytes. Here we review the major cell populations thought to be endogenous cardiac stem cells.

### Isl-1+ cardiac stem cells

Progenitor cells of the first and second heart fields in the developing heart depend on cardiac-specific transcription factors for their differentiation. Islet-1 (Isl-1) is a marker of cardiac progenitors arising from the second heart field (Klaus et al. 2012). Substantial work using murine models of cardiogenesis have reported the progenitor phenotype of this population (Laugwitz et al. 2005; Moretti et al. 2006). Furthermore, in a report by Bu et al., transgenic and gene-targeting approaches in human embryonic stem cell lines demonstrated that purified Isl-1+ CSCs are pluripotent, revealing their ability to form cardiomyocytes, endothelial cells, vascular smooth muscle and cardiac conduction tissue (Bu et al. 2009). This population of cells was proven to be distinct from other CSC populations (c-kit, Sca-1 or side-population cells ‒ see below) (Laugwitz et al. 2005).

### c-kit + cardiac stem cells

c-kit is a tyrosine kinase surface receptor originally shown to enrich for HSCs. It has now been used to identify stem cell populations in other organs including the heart. Building on their previous study involving sex-mismatched cardiac transplants in humans (Quaini et al. 2002), the same laboratory proposing the presence of endogenous CSCs was able to identify c-kit + CSCs in the hearts of dogs (Linke et al. 2005), mice (Urbanek et al. 2005) and humans (Bearzi et al. 2007). In rats, they found that c-kit + CSCs formed new vasculature and immature myocytes when injected into an infarcted heart (Beltrami et al. 2003), subsequently improving cardiac function. The c-kit + CSCs formed clusters in the interstitia between myocytes and demonstrated signs of early cardiac myogenic differentiation evidenced by expression of the transcription factors *Gata-4, Nkx2-5 and Mef2c*. Furthermore, after in vitro expansion, these cells showed typical characteristics of stem cells, including long term self-renewal, clonogenicity and multipotency.

It is important to note that controversy surrounds the myogenic potential of c-kit + CSCs. In a study by Zaruba et al., the cardiomyogenic ability of c-kit + CSCs was high in neonatal mice but decreased significantly with age and was negligible by adulthood (Zaruba et al. 2010). These findings are supported by other studies, including that by Fazel et al., which also reported that c-kit + CSCs were actually of extra-cardiac origin (Fazel et al. 2006). Before inducing MI in mice, Fazel et al. found that c-kit + cardiac cells were rare (a finding also shared by others (Chong et al. 2011; Beltrami et al. 2003)) and 1 month after MI, none of the c-kit + cells were cardiomyocytes. They also found that approximately 74% of c-kit + cardiac cells after MI were in fact BM-derived. Contrasting these reports, a recent transcriptional profiling study by Dey et al. reported a clear difference in the molecular signatures of c-kit + CSCs and c-kit + HSCs (Dey et al. 2013), suggesting different origins and cell fates of BM and cardiac c-kit + cells. Very recently, Berlo et al. used multiple rigorous fate mapping approaches in genetically modified mice to prove that negligible cardiomyocyte contribution occurs from endogenous cardiac c-kit + populations after normal ageing or cardiac injury (van Berlo et al. 2014).

Despite the debate about c-kit + CSCs in preclinical models, the Stem Cell Infusion in Patients with Ischemic Cardiomyopathy (SCIPIO) phase 1 clinical trial proceeded unabated and is currently ongoing (Bolli et al. 2011; Chugh et al. 2012) (Table 2). In this trial, autologous c-kit + CSCs were isolated and expanded ex vivo from patients undergoing coronary artery bypass surgery after MI. Participants were then randomized to receive either intracoronary infusion of these c-kit + CSCs or conventional therapy. Preliminary results of this study showed that isolation and expansion of c-kit + CSCs in this manner was feasible and that subsequent intracoronary delivery did not compromise patient safety (Chugh et al. 2012). Furthermore, a significant improvement in both global and regional LV function and a reduction in infarct size were observed in the c-kit + CSC group when compared to conventional therapy (Bolli et al. 2011). It is important to note that concerns regarding the integrity of certain data generated during the trial have been raised by the Lancet editors (The Lancet Editors 2014) and that concerns regarding patient randomization have also been raised (Nowbar et al. 2014).

**Table 2:** **Recent and ongoing clinical trials involving endogenous cardiac stem cells in patients with ischemic cardiomyopathy**

Trial name/Investigator	Study identifier	Comparators	Endpoint	Patients	Delivery route	Type classification
**Cardiosphere-derived Stem Cells**						
ALLSTAR	NCT01458405	Allogeneic CDCs vs placebo	Infarct size	274	Intracoronary	Safety + efficacy, Phase 1/2
CADUCEUS*	NCT00893360	CDCs (12.5 or 25 million) vs no intervention	Safety	31	Intracoronary	Safety, Phase 1
**Cardiac Stem Cells + bFGF**						
ALCADIA*	NCT00981006	CSCs + bFGF infusion	Safety	6	Intramyocardial	Safety, Phase 1
Cardiac Stem Cells (undefined)						
Vakilian et al.	NCT01758406	CSCs vs placebo	Mortality + LVEF	50	Intracoronary	Safety + efficacy, Phase 2
SCIPIO*	NCT00474461	CSCs infusion	SAE	40	Intracoronary	Safety + efficacy, Phase 1

All trials use autologous infusions unless otherwise stated. Abbreviations: bFGF-Basic Fibroblast Growth Factor, CDCs-Cardiosphere-derived Cells, CSCs-Cardiac Stem Cells, LVEF-Left Ventricular Ejection Fraction, SAE-Serious Adverse Events.

* Denotes trials with published results (including preliminary results).

### Cardiosphere-derived cardiac stem cells

The term “cardiosphere” was first coined by Messina et al. describing a population of undifferentiated cells, isolated from cardiac biopsies and obtained by enzymatic digestion of explanted cardiac tissue (Messina et al. 2004). Cardiospheres (CSs) and cardiosphere-derived cells (CDCs) have since been characterized as a heterogenous cell population, containing a central core of c-kit + cells which are surrounded by other cells including those expressing the stromal cell marker CD105. In early preclinical work, CDCs injected into infarcted murine hearts improved cardiac function via apparent differentiation into both cardiomyocytes and vasculature (Messina et al. 2004). Subsequently, a more clinically applicable approach was devised for the isolation and expansion of human CDCs from endomyocardial biopsy specimens (Smith et al. 2007). The authors of this study reported similar findings regarding the cardiac regenerative potential of CDCs after engrafting the cells into infarcted hearts of immunocompromised mice. Techniques later used for the isolation and administration of human CDCs post-MI in clinical trials (see below) were refined using a porcine model of myocardial ischemia/reperfusion (Johnston et al. 2009).

In contrast to the findings above, Andersen et al. used novel culture methods to contest the cardiomyogenic differentiation potential of human CSs (Andersen et al. 2009). They claimed that contamination with myocardial tissue fragments is likely to be responsible for the ability of CSs (isolated from neonatal rats) to spontaneously contract. However, further studies (Davis et al. 2009, 2010) have refuted these results, thereby re-establishing CDCs as a promising cardiac cell therapy candidate.

A recent study by Li et al. compared the functional benefits of CDCs to those of enriched c-kit + CSCs, BM-MNCs, BM-MSCs, and AD-MSCs (Li et al. 2012). They found that CDCs had superior potency and myocardial repair efficacy in murine models compared to the other stem cell populations. This suggests that cell interactions amongst the heterogenous CDC cell mix may be advantageous. The model of allogeneic rather than autologous CDC delivery in cardiac cell therapy has also been validated (Malliaras et al. 2012). This further expands their prospective therapeutic potential by enabling an “off the shelf” product.

In addition to c-kit + CSCs, CDCs are the only other CSC to have published results from clinical trials. CADUCEUS (Cardiosphere-Derived Autologous Stem Cells to Reverse ventricular dysfunction) was a prospective, randomized phase 1 trial assessing the safety of autologous intracoronary CDC delivery in patients post-MI (Makkar et al. 2012). Investigators of this study utilized percutaneous techniques to obtain endomyocardial biopsies from which CDCs were isolated and expanded ex vivo. In a later procedure, 2–4 weeks after MI, the expanded CDCs were delivered by intracoronary injection into the infarct-related arteries. Results from the CADUCEUS study show that CDC therapy appears to be safe. Magnetic resonance imaging revealed that at 3 months, CDC treatment reduced scar mass, increased viable heart mass as well as increased regional systolic wall thickening when compared to controls (Makkar et al. 2012). Notably a statistically significant improvement in overall LV function was not reported. Nevertheless, these results have encouraged further investigation of allogeneic CDC therapy in the larger ALLSTAR clinical trial (NCT01458405) (Table 2).

### Side-population cardiac progenitors

The cardiac side-population (SP) represents a subpopulation of cardiac progenitors possessing the unique ability to efflux a DNA binding dye (namely Hoechst 33342) conferred by an ATP-binding cassette transporter (Unno et al. 2012). In humans, these protein transporters are encoded by the *ABCG2* gene, which can be used as a determinant of the SP cell phenotype (Martin et al. 2004). SP cells comprise 4%, 2% and 1.2% of cells in the fetal, neonatal, and adult rat heart, respectively (Leri et al. 2011). Initial reports by Hierlihy et al. indicate that cardiac SP cells (C-SPs) display stem cell activity, lack markers of differentiated cell lineages, and possess significant cardiomyogenic potential in vitro (Hierlihy et al. 2002). Confirming the latter findings, Martin et al. found that C-SPs expressed α-ACTININ (myocyte protein) when co-cultured with cardiac main population cells (Martin et al. 2004), indicating possible cardiomyocyte differentiation potential.

Similar to other CSC populations, the origin of C-SPs has been questioned. To address a possible BM origin for C-SPs, Mouquet et al. transplanted fluorescently labeled BM into wild-type adult mice (Mouquet et al. 2005) and investigated the effect of MI on these cells. Injured hearts demonstrated an acute depletion of C-SPs following MI, which was replenished (by up to 25%) by fluorescently labeled BM-derived stem cells within 7 days. These cells then proceeded to adopt a C-SP phenotype, suggesting that a significant portion of the C-SP population is in fact BM-derived, rather than solely of cardiac origin. Combining cell surface marker studies with lineage tracing experiments will enable more accurate tracking of C-SP origins and cell fates in vivo, allowing for better characterization of their cardiac regenerative abilities.

### Sca-1+ cardiac stem cells

Stem cell antigen-1 (Sca-1) is widely used to enrich HSCs in mice (Holmes and Stanford 2007). Humans lack the murine Sca-1 gene and it is currently unknown whether there is a true human orthologue. Sca-1+ CSCs were first characterized in the adult murine myocardium by Oh et al. as cardiac stem/progenitor cells lacking hematopoietic lineage markers (Oh et al. 2003). Prior to stimulation with 5-AZA, freshly isolated Sca-1+ CSCs expressed the cardiogenic transcription factors *Gata-4, Mef2c and Tef-1*. Upon stimulation, a small percentage of these cells began to express cardiac structural genes (sarcomeric α-actin, *Tnni3, Nkx2-5, β-Mhc and α-Mhc*) and consequently acquired a phenotype resembling that of cardiomyocytes. The authors reported that intravenously delivered Sca-1+ CSCs home to sites of tissue injury and form new cardiomyocytes in mice post-MI, presenting a more clinically applicable view of their regenerative potential.

Matsuura et al. subsequently demonstrated Sca-1+ CSC multipotency by showing differentiation into osteocytes and adipocytes (Matsuura et al. 2004). Furthermore, they demonstrated that refined in vitro procedures using oxytocin instead of 5-AZA to stimulate Sca-1+ CSC differentiation resulted in the generation of spontaneously beating cardiomyocytes. This added weight to the evidence supporting the in vitro cardiac differentiation capacity of Sca-1+ CSCs. Further experimentation by Wang et al. revealed that it is in fact the Sca-1+/CD31- sub-population that possesses cardiomyogenic potential (Wang et al. 2006).

Notably, murine transgenic technology has provided valuable insight into the molecular role of Sca-1+ CSCs. Tateishi et al. demonstrated that knockdown of Sca-1 transcripts in CSCs led to retarded ex vivo expansion and cell apoptosis (through Akt inactivation) (Tateishi et al. 2007). Their results also show that cardiomyocytes require Sca-1 to upregulate secretion of paracrine effectors which induce angiogenesis and limit cardiac apoptosis. This implies that therapeutically, Sca-1 may promote CSC survival following engraftment into injured tissue and will in turn influence revascularization and cardiac repair.

### Epicardium derived cells

The epicardium is the outer layer of heart and inner layer of the pericardium, consisting predominantly of mesothelial cells and dense connective tissue. Epicardium-derived cells (EPDCs) have long been known to play a fundamental role in the developing embryonic heart. Although these cells were thought to be quiescent in healthy adult hearts, recent evidence suggests that injury-associated signals after MI induce reactivation of EPDCs, promoting heart regeneration and injury reduction (Zhou et al. 2011). Another study has described this process as epithelial to mesenchymal transformation involving the migration of multipotent EPDCs from the epicardium to the subepicardial matrix, subsequently forming coronary vasculature and cells of the cardiac interstitium (van Tuyn et al. 2007). Paracrine mediators secreted by EPDCs have been shown to underlie their regenerative potential (Zhou et al. 2011); however, their direct contribution to the cardiomyocyte and endothelial cell lineages remains controversial (Wessels and Perez-Pomares 2004).

Smart et al. recently demonstrated that preconditioning of adult murine hearts with thymosin β4 (crucial for neovascularization of the neonatal heart), prior to infarction, significantly activated the quiescent epicardium (Smart et al. 2007). Activation led to EPDC mobilization and neovascularization of the heart following MI, suggesting that the adult mammalian epicardium harbors significant regenerative potential. These recent studies highlight the therapeutic potential of multipotent EPDCs found in the postnatal mammalian heart.

### Sca-1+/PDGFRα + cardiac stem cells

Amongst the various Sca-1+ subpopulations, a study by Chong et al. identified a stem cell population that co-expresses Sca-1 and platelet derived growth factor receptor α (PDGFRα) (Chong et al. 2011). By comprehensive lineage tracing experiments in genetic mouse models, this CSC population was found to originate from the embryonic epicardium (Chong et al. 2011) and exhibited the cardinal features of stem cells (self-renewal, clonogenicity and multipotency). Similar to BM-MSCs, Sca-1+/PDGFRα + CSCs can form clonal colonies and were therefore called cardiac colony-forming unit fibrolasts (cCFU-Fs). Recently, the same CSC population described in these murine studies has been identified in human hearts (Chong et al. 2013). It is likely that cCFU-Fs contribute to the regenerative features observed in EPDCs, but further verification is required to characterize the extent and mechanism of their contribution to cardiac regeneration of injured hearts.

## Delivery

No agreement has been reached regarding the optimum method of delivery of transplanted cells; intramyocardial (IM), intravenous (IV) and intracoronary (IC) delivery methods are all used interchangeably. The most clinically practiced form of cell delivery is the IC approach which provides a direct route of cell delivery via coronary arteries to myocardial sites of interest (Sheng et al. 2013). This procedure is normally carried out simultaneously during percutaneous coronary intervention post-MI. Whilst being safe and efficient, 1 major limitation of this procedure is that cells are not able to reach areas of myocardium that are poorly perfused.

IV injections are only used selectively in patients following MI, as they rely on physiological homing signals from injured heart tissue, a state not present in chronic heart failure. Although widely inefficient, IV infusions are simple and minimally invasive. In contrast, transepicardial IM delivery is significantly invasive but provides the most direct and precise delivery method. Transendocardial IM delivery via percutaneous delivery catheters has been developed as possibly the most efficient yet least invasive delivery method. The AlsterMACS (Intracoronary Versus Intramyocardial Application of Enriched CD133pos Autologous Bone Marrow Derived Stem Cells) trial (NCT01337011) compares 2 of the above delivery techniques, IC and IM, in order to establish a universal mechanism for the administration of stem cells to patients with ischemic heart disease (Table 1). Another trial run by Vrtovec et al. states through preliminary results that transendocardial IM cell transplantation is associated with higher retention rates and greater improvement in ventricular function compared with IC administration (Vrtovec et al. 2013) (NCT01350310).

In addition to conventional delivery methods, various tissue engineering techniques provide novel approaches to improve sustainability and accuracy of cell culture transplantation, augmenting the effects of transplanted cells.

## Summary and prospects

The long held view of the human adult heart as a quiescent organ incapable of regeneration has only recently been successfully challenged. It is now clear that the heart possesses a small but significant ability to regenerate after insults such as MI. Stem Cell therapy can enhance this ability and may ultimately provide a viable clinical therapy to treat post-MI cardiac dysfunction. However, considerable work remains. Fore mostly, the best candidate cell type needs to be elucidated. This will likely become clear with time. In addition, the mechanisms underpinning favorable cardiac effects of most, if not all of the stem cells reviewed, are incompletely understood. Although not a prerequisite for clinical therapy, mechanistic understanding will aid further refinement of cardiac cell therapy and will speed effective clinical translation. We strongly believe that cardiac regeneration, either by delivery of extra-cardiac stem cells or by enhancing endogenous mechanisms, will change the future of MI treatment.

## References

[CR1] Abdel-Latif A, Bolli R, Tleyjeh IM, Montori VM, Perin EC, Hornung CA, Zuba-Surma EK, Al-Mallah M, Dawn B (2007). Adult bone marrow-derived cells for cardiac repair: a systematic review and meta-analysis. Arch Intern Med.

[CR2] Aggarwal S, Pittenger MF (2005). Human mesenchymal stem cells modulate allogeneic immune cell responses. Blood.

[CR3] Andersen DC, Andersen P, Schneider M, Jensen HB, Sheikh SP (2009). Murine “cardiospheres” are not a source of stem cells with cardiomyogenic potential. Stem Cells.

[CR4] Asahara T, Murohara T, Sullivan A, Silver M, van der Zee R, Li T, Witzenbichler B, Schatteman G, Isner JM (1997). Isolation of putative progenitor endothelial cells for angiogenesis. Science.

[CR5] Balsam LB, Wagers AJ, Christensen JL, Kofidis T, Weissman IL, Robbins RC (2004). Haematopoietic stem cells adopt mature haematopoietic fates in ischaemic myocardium. Nature.

[CR6] Bartunek J, Behfar A, Dolatabadi D, Vanderheyden M, Ostojic M, Dens J, El Nakadi B, Banovic M, Beleslin B, Vrolix M, Legrand V, Vrints C, Vanoverschelde JL, Crespo-Diaz R, Homsy C, Tendera M, Waldman S, Wijns W, Terzic A (2013). Cardiopoietic stem cell therapy in heart failure: the C-CURE (Cardiopoietic stem Cell therapy in heart failURE) multicenter randomized trial with lineage-specified biologics. J Am Coll Cardiol.

[CR7] Bearzi C, Rota M, Hosoda T, Tillmanns J, Nascimbene A, De Angelis A, Yasuzawa-Amano S, Trofimova I, Siggins RW, Lecapitaine N, Cascapera S, Beltrami AP, D’Alessandro DA, Zias E, Quaini F, Urbanek K, Michler RE, Bolli R, Kajstura J, Leri A, Anversa P (2007). Human cardiac stem cells. Proc Natl Acad Sci U S A.

[CR8] Beltrami AP, Barlucchi L, Torella D, Baker M, Limana F, Chimenti S, Kasahara H, Rota M, Musso E, Urbanek K, Leri A, Kajstura J, Nadal-Ginard B, Anversa P (2003). Adult cardiac stem cells are multipotent and support myocardial regeneration. Cell.

[CR9] Bolli R, Chugh AR, D’Amario D, Loughran JH, Stoddard MF, Ikram S, Beache GM, Wagner SG, Leri A, Hosoda T, Sanada F, Elmore JB, Goichberg P, Cappetta D, Solankhi NK, Fahsah I, Rokosh DG, Slaughter MS, Kajstura J, Anversa P (2011). Cardiac stem cells in patients with ischaemic cardiomyopathy (SCIPIO): initial results of a randomised phase 1 trial. Lancet.

[CR10] Bu L, Jiang X, Martin-Puig S, Caron L, Zhu S, Shao Y, Roberts DJ, Huang PL, Domian IJ, Chien KR (2009). Human ISL1 heart progenitors generate diverse multipotent cardiovascular cell lineages. Nature.

[CR11] Cai L, Johnstone BH, Cook TG, Tan J, Fishbein MC, Chen PS, March KL (2009). IFATS collection: human adipose tissue-derived stem cells induce angiogenesis and nerve sprouting following myocardial infarction, in conjunction with potent preservation of cardiac function. Stem Cells.

[CR12] Challen GA, Boles NC, Chambers SM, Goodell MA (2010). Distinct hematopoietic stem cell subtypes are differentially regulated by TGF-beta1. Cell Stem Cell.

[CR13] Choi WY, Poss KD (2012). Cardiac regeneration. Curr Top Dev Biol.

[CR14] Chong JJH, Murry CE (2014). Cardiac regeneration using pluripotent stem cells–progression to large animal models. Stem Cell Res.

[CR15] Chong JJ, Chandrakanthan V, Xaymardan M, Asli NS, Li J, Ahmed I, Heffernan C, Menon MK, Scarlett CJ, Rashidianfar A, Biben C, Zoellner H, Colvin EK, Pimanda JE, Biankin AV, Zhou B, Pu WT, Prall OW, Harvey RP (2011). Adult cardiac-resident MSC-like stem cells with a proepicardial origin. Cell Stem Cell.

[CR16] Chong JJ, Reinecke H, Iwata M, Torok-Storb B, Stempien-Otero A, Murry CE (2013). Progenitor cells identified by PDGFR-alpha expression in the developing and diseased human heart. Stem Cells Dev.

[CR17] Chong JJH, Yang X, Don CW, Minami E, Liu Y-W, Weyers JJ, Mahoney WM, Van Biber B, Cook SM, Palpant NJ, Gantz JA, Fugate JA, Muskheli V, Gough GM, Vogel KW, Astley CA, Hotchkiss CE, Baldessari A, Pabon L, Reinecke H, Gill EA, Nelson V, Kiem H-P, Laflamme MA, Murry CE (2014). Human embryonic-stem-cell-derived cardiomyocytes regenerate non-human primate hearts. Nature.

[CR18] Chugh AR, Beache GM, Loughran JH, Mewton N, Elmore JB, Kajstura J, Pappas P, Tatooles A, Stoddard MF, Lima JA, Slaughter MS, Anversa P, Bolli R (2012). Administration of cardiac stem cells in patients with ischemic cardiomyopathy: the SCIPIO trial: surgical aspects and interim analysis of myocardial function and viability by magnetic resonance. Circulation.

[CR19] Clifford DM, Fisher SA, Brunskill SJ, Doree C, Mathur A, Clarke MJ, Watt SM, Martin-Rendon E (2012). Long-term effects of autologous bone marrow stem cell treatment in acute myocardial infarction: factors that may influence outcomes. PLoS One.

[CR20] da Silva Meirelles L, Chagastelles PC, Nardi NB (2006). Mesenchymal stem cells reside in virtually all post-natal organs and tissues. J Cell Sci.

[CR21] Davis DR, Zhang Y, Smith RR, Cheng K, Terrovitis J, Malliaras K, Li TS, White A, Makkar R, Marban E (2009). Validation of the cardiosphere method to culture cardiac progenitor cells from myocardial tissue. PLoS One.

[CR22] Davis DR, Ruckdeschel Smith R, Marban E (2010). Human cardiospheres are a source of stem cells with cardiomyogenic potential. Stem Cells.

[CR23] Dey D, Han L, Bauer M, Sanada F, Oikonomopoulos A, Hosoda T, Unno K, De Almeida P, Leri A, Wu JC (2013). Dissecting the molecular relationship among various cardiogenic progenitor cells. Circ Res.

[CR24] Di Nicola M, Carlo-Stella C, Magni M, Milanesi M, Longoni PD, Matteucci P, Grisanti S, Gianni AM (2002). Human bone marrow stromal cells suppress T-lymphocyte proliferation induced by cellular or nonspecific mitogenic stimuli. Blood.

[CR25] Eckfeldt CE, Mendenhall EM, Verfaillie CM (2005). The molecular repertoire of the ‘almighty’ stem cell. Nat Rev Mol Cell Biol.

[CR26] Fazel S, Cimini M, Chen L, Li S, Angoulvant D, Fedak P, Verma S, Weisel RD, Keating A, Li RK (2006). Cardioprotective c-kit + cells are from the bone marrow and regulate the myocardial balance of angiogenic cytokines. J Clin Invest.

[CR27] Fernandes S, Naumova AV, Zhu WZ, Laflamme MA, Gold J, Murry CE (2010). Human embryonic stem cell-derived cardiomyocytes engraft but do not alter cardiac remodeling after chronic infarction in rats. J Mol Cell Cardiol.

[CR28] Friedenstein AJ, Chailakhjan RK, Lalykina KS (1970). The development of fibroblast colonies in monolayer cultures of guinea-pig bone marrow and spleen cells. Cell Tissue Kinet.

[CR29] Friis T, Haack-Sorensen M, Mathiasen AB, Ripa RS, Kristoffersen US, Jorgensen E, Hansen L, Bindslev L, Kjaer A, Hesse B, Dickmeiss E, Kastrup J (2011). Mesenchymal stromal cell derived endothelial progenitor treatment in patients with refractory angina. Scand Cardiovasc J.

[CR30] Hare JM, Traverse JH, Henry TD, Dib N, Strumpf RK, Schulman SP, Gerstenblith G, DeMaria AN, Denktas AE, Gammon RS, Hermiller JB, Reisman MA, Schaer GL, Sherman W (2009). A randomized, double-blind, placebo-controlled, dose-escalation study of intravenous adult human mesenchymal stem cells (prochymal) after acute myocardial infarction. J Am Coll Cardiol.

[CR31] Hare JM, Fishman JE, Gerstenblith G, DiFede Velazquez DL, Zambrano JP, Suncion VY, Tracy M, Ghersin E, Johnston PV, Brinker JA, Breton E, Davis-Sproul J, Schulman IH, Byrnes J, Mendizabal AM, Lowery MH, Rouy D, Altman P, Wong Po Foo C, Ruiz P, Amador A, Da Silva J, McNiece IK, Heldman AW, George R, Lardo A (2012). Comparison of allogeneic vs autologous bone marrow-derived mesenchymal stem cells delivered by transendocardial injection in patients with ischemic cardiomyopathy: the POSEIDON randomized trial. JAMA.

[CR32] Heldman AW, DiFede DL, Fishman JE, Zambrano JP, Trachtenberg BH, Karantalis V, Mushtaq M, Williams AR, Suncion VY, McNiece IK, Ghersin E, Soto V, Lopera G, Miki R, Willens H, Hendel R, Mitrani R, Pattany P, Feigenbaum G, Oskouei B, Byrnes J, Lowery MH, Sierra J, Pujol MV, Delgado C, Gonzalez PJ, Rodriguez JE, Bagno LL, Rouy D, Altman P (2014). Transendocardial mesenchymal stem cells and mononuclear bone marrow cells for ischemic cardiomyopathy: the TAC-HFT randomized trial. JAMA.

[CR33] Hierlihy AM, Seale P, Lobe CG, Rudnicki MA, Megeney LA (2002). The post-natal heart contains a myocardial stem cell population. FEBS Lett.

[CR34] Holmes C, Stanford WL (2007). Concise review: stem cell antigen-1: expression, function, and enigma. Stem Cells.

[CR35] Johnston PV, Sasano T, Mills K, Evers R, Lee ST, Smith RR, Lardo AC, Lai S, Steenbergen C, Gerstenblith G, Lange R, Marban E (2009). Engraftment, differentiation, and functional benefits of autologous cardiosphere-derived cells in porcine ischemic cardiomyopathy. Circulation.

[CR36] Karantalis V, DiFede DL, Gerstenblith G, Pham S, Symes J, Zambrano JP, Fishman J, Pattany P, McNiece I, Conte J, Schulman S, Wu K, Shah A, Breton E, Davis-Sproul J, Schwarz R, Feigenbaum G, Mushtaq M, Suncion VY, Lardo AC, Borrello I, Mendizabal A, Karas TZ, Byrnes J, Lowery M, Heldman AW, Hare JM (2014). Autologous mesenchymal stem cells produce concordant improvements in regional function, tissue perfusion, and fibrotic burden when administered to patients undergoing coronary artery bypass grafting: the Prospective Randomized Study of Mesenchymal Stem Cell Therapy in Patients Undergoing Cardiac Surgery (PROMETHEUS) trial. Circ Res.

[CR37] Klaus A, Muller M, Schulz H, Saga Y, Martin JF, Birchmeier W (2012). Wnt/beta-catenin and Bmp signals control distinct sets of transcription factors in cardiac progenitor cells. Proc Natl Acad Sci U S A.

[CR38] Laflamme MA, Murry CE (2011). Heart regeneration. Nature.

[CR39] Laflamme MA, Chen KY, Naumova AV, Muskheli V, Fugate JA, Dupras SK, Reinecke H, Xu C, Hassanipour M, Police S, O’Sullivan C, Collins L, Chen Y, Minami E, Gill EA, Ueno S, Yuan C, Gold J, Murry CE (2007). Cardiomyocytes derived from human embryonic stem cells in pro-survival factors enhance function of infarcted rat hearts. Nat Biotechnol.

[CR40] Laugwitz KL, Moretti A, Lam J, Gruber P, Chen Y, Woodard S, Lin LZ, Cai CL, Lu MM, Reth M, Platoshyn O, Yuan JX, Evans S, Chien KR (2005). Postnatal isl1+ cardioblasts enter fully differentiated cardiomyocyte lineages. Nature.

[CR41] Leobon B, Garcin I, Menasche P, Vilquin JT, Audinat E, Charpak S (2003). Myoblasts transplanted into rat infarcted myocardium are functionally isolated from their host. Proc Natl Acad Sci U S A.

[CR42] Leri A, Kajstura J, Anversa P (2011). Role of cardiac stem cells in cardiac pathophysiology: a paradigm shift in human myocardial biology. Circ Res.

[CR43] Li X, Yu X, Lin Q, Deng C, Shan Z, Yang M, Lin S (2007). Bone marrow mesenchymal stem cells differentiate into functional cardiac phenotypes by cardiac microenvironment. J Mol Cell Cardiol.

[CR44] Li H, Zuo S, He Z, Yang Y, Pasha Z, Wang Y, Xu M (2010). Paracrine factors released by GATA-4 overexpressed mesenchymal stem cells increase angiogenesis and cell survival. Am J Physiol Heart Circ Physiol.

[CR45] Li TS, Cheng K, Malliaras K, Smith RR, Zhang Y, Sun B, Matsushita N, Blusztajn A, Terrovitis J, Kusuoka H, Marban L, Marban E (2012). Direct comparison of different stem cell types and subpopulations reveals superior paracrine potency and myocardial repair efficacy with cardiosphere-derived cells. J Am Coll Cardiol.

[CR46] Linke A, Muller P, Nurzynska D, Casarsa C, Torella D, Nascimbene A, Castaldo C, Cascapera S, Bohm M, Quaini F, Urbanek K, Leri A, Hintze TH, Kajstura J, Anversa P (2005). Stem cells in the dog heart are self-renewing, clonogenic, and multipotent and regenerate infarcted myocardium, improving cardiac function. Proc Natl Acad Sci U S A.

[CR47] Makkar RR, Smith RR, Cheng K, Malliaras K, Thomson LE, Berman D, Czer LS, Marban L, Mendizabal A, Johnston PV, Russell SD, Schuleri KH, Lardo AC, Gerstenblith G, Marban E (2012). Intracoronary cardiosphere-derived cells for heart regeneration after myocardial infarction (CADUCEUS): a prospective, randomised phase 1 trial. Lancet.

[CR48] Malliaras K, Li TS, Luthringer D, Terrovitis J, Cheng K, Chakravarty T, Galang G, Zhang Y, Schoenhoff F, Van Eyk J, Marban L, Marban E (2012). Safety and efficacy of allogeneic cell therapy in infarcted rats transplanted with mismatched cardiosphere-derived cells. Circulation.

[CR49] Martin CM, Meeson AP, Robertson SM, Hawke TJ, Richardson JA, Bates S, Goetsch SC, Gallardo TD, Garry DJ (2004). Persistent expression of the ATP-binding cassette transporter, Abcg2, identifies cardiac SP cells in the developing and adult heart. Dev Biol.

[CR50] Martin-Rendon E, Brunskill SJ, Hyde CJ, Stanworth SJ, Mathur A, Watt SM (2008). Autologous bone marrow stem cells to treat acute myocardial infarction: a systematic review. Eur Heart J.

[CR51] Matsuura K, Nagai T, Nishigaki N, Oyama T, Nishi J, Wada H, Sano M, Toko H, Akazawa H, Sato T, Nakaya H, Kasanuki H, Komuro I (2004). Adult cardiac Sca-1-positive cells differentiate into beating cardiomyocytes. J Biol Chem.

[CR52] Menasche P, Hagege AA, Vilquin JT, Desnos M, Abergel E, Pouzet B, Bel A, Sarateanu S, Scorsin M, Schwartz K, Bruneval P, Benbunan M, Marolleau JP, Duboc D (2003). Autologous skeletal myoblast transplantation for severe postinfarction left ventricular dysfunction. J Am Coll Cardiol.

[CR53] Menasche P, Alfieri O, Janssens S, McKenna W, Reichenspurner H, Trinquart L, Vilquin JT, Marolleau JP, Seymour B, Larghero J, Lake S, Chatellier G, Solomon S, Desnos M, Hagege AA (2008). The Myoblast Autologous Grafting in Ischemic Cardiomyopathy (MAGIC) trial: first randomized placebo-controlled study of myoblast transplantation. Circulation.

[CR54] Messina E, De Angelis L, Frati G, Morrone S, Chimenti S, Fiordaliso F, Salio M, Battaglia M, Latronico MV, Coletta M, Vivarelli E, Frati L, Cossu G, Giacomello A (2004). Isolation and expansion of adult cardiac stem cells from human and murine heart. Circ Res.

[CR55] Meyer GP, Wollert KC, Lotz J, Steffens J, Lippolt P, Fichtner S, Hecker H, Schaefer A, Arseniev L, Hertenstein B, Ganser A, Drexler H (2006). Intracoronary bone marrow cell transfer after myocardial infarction: eighteen months’ follow-up data from the randomized, controlled BOOST (BOne marrOw transfer to enhance ST-elevation infarct regeneration) trial. Circulation.

[CR56] Min JY, Yang Y, Converso KL, Liu L, Huang Q, Morgan JP, Xiao YF (2002). Transplantation of embryonic stem cells improves cardiac function in postinfarcted rats. J Appl Physiol.

[CR57] Moretti A, Caron L, Nakano A, Lam JT, Bernshausen A, Chen Y, Qyang Y, Bu L, Sasaki M, Martin-Puig S, Sun Y, Evans SM, Laugwitz KL, Chien KR (2006). Multipotent embryonic isl1+ progenitor cells lead to cardiac, smooth muscle, and endothelial cell diversification. Cell.

[CR58] Mouquet F, Pfister O, Jain M, Oikonomopoulos A, Ngoy S, Summer R, Fine A, Liao R (2005). Restoration of cardiac progenitor cells after myocardial infarction by self-proliferation and selective homing of bone marrow-derived stem cells. Circ Res.

[CR59] Murry CE, Soonpaa MH, Reinecke H, Nakajima H, Nakajima HO, Rubart M, Pasumarthi KB, Virag JI, Bartelmez SH, Poppa V, Bradford G, Dowell JD, Williams DA, Field LJ (2004). Haematopoietic stem cells do not transdifferentiate into cardiac myocytes in myocardial infarcts. Nature.

[CR60] Nadal-Ginard B, Kajstura J, Leri A, Anversa P (2003). Myocyte death, growth, and regeneration in cardiac hypertrophy and failure. Circ Res.

[CR61] Nowbar AN, Mielewczik M, Karavassilis M, Dehbi H-M, Shun-Shin MJ, Jones S, Howard JP, Cole GD, Francis DP (2014). Discrepancies in autologous bone marrow stem cell trials and enhancement of ejection fraction (DAMASCENE): weighted regression and meta-analysis.

[CR62] Oh H, Bradfute SB, Gallardo TD, Nakamura T, Gaussin V, Mishina Y, Pocius J, Michael LH, Behringer RR, Garry DJ, Entman ML, Schneider MD (2003). Cardiac progenitor cells from adult myocardium: homing, differentiation, and fusion after infarction. Proc Natl Acad Sci U S A.

[CR63] Orlic D, Kajstura J, Chimenti S, Jakoniuk I, Anderson SM, Li B, Pickel J, McKay R, Nadal-Ginard B, Bodine DM, Leri A, Anversa P (2001). Bone marrow cells regenerate infarcted myocardium. Nature.

[CR64] Orn S, Manhenke C, Anand IS, Squire I, Nagel E, Edvardsen T, Dickstein K (2007). Effect of left ventricular scar size, location, and transmurality on left ventricular remodeling with healed myocardial infarction. Am J Cardiol.

[CR65] Pearl JI, Lee AS, Leveson-Gower DB, Sun N, Ghosh Z, Lan F, Ransohoff J, Negrin RS, Davis MM, Wu JC (2011). Short-term immunosuppression promotes engraftment of embryonic and induced pluripotent stem cells. Cell Stem Cell.

[CR66] Perin EC, Willerson JT, Pepine CJ, Henry TD, Ellis SG, Zhao DX, Silva GV, Lai D, Thomas JD, Kronenberg MW, Martin AD, Anderson RD, Traverse JH, Penn MS, Anwaruddin S, Hatzopoulos AK, Gee AP, Taylor DA, Cogle CR, Smith D, Westbrook L, Chen J, Handberg E, Olson RE, Geither C, Bowman S, Francescon J, Baraniuk S, Piller LB, Simpson LM (2012). Effect of transendocardial delivery of autologous bone marrow mononuclear cells on functional capacity, left ventricular function, and perfusion in chronic heart failure: the FOCUS-CCTRN trial. JAMA.

[CR67] Quaini F, Urbanek K, Beltrami AP, Finato N, Beltrami CA, Nadal-Ginard B, Kajstura J, Leri A, Anversa P (2002). Chimerism of the transplanted heart. N Engl J Med.

[CR68] Rangappa S, Entwistle JW, Wechsler AS, Kresh JY (2003). Cardiomyocyte-mediated contact programs human mesenchymal stem cells to express cardiogenic phenotype. J Thorac Cardiovasc Surg.

[CR69] Rasmussen TL, Raveendran G, Zhang J, Garry DJ (2011). Getting to the heart of myocardial stem cells and cell therapy. Circulation.

[CR70] Roell W, Lewalter T, Sasse P, Tallini YN, Choi BR, Breitbach M, Doran R, Becher UM, Hwang SM, Bostani T, von Maltzahn J, Hofmann A, Reining S, Eiberger B, Gabris B, Pfeifer A, Welz A, Willecke K, Salama G, Schrickel JW, Kotlikoff MI, Fleischmann BK (2007). Engraftment of connexin 43-expressing cells prevents post-infarct arrhythmia. Nature.

[CR71] Roger VL, Go AS, Lloyd-Jones DM, Adams RJ, Berry JD, Brown TM, Carnethon MR, Dai S, de Simone G, Ford ES, Fox CS, Fullerton HJ, Gillespie C, Greenlund KJ, Hailpern SM, Heit JA, Ho PM, Howard VJ, Kissela BM, Kittner SJ, Lackland DT, Lichtman JH, Lisabeth LD, Makuc DM, Marcus GM, Marelli A, Matchar DB, McDermott MM, Meigs JB, Moy CS (2011). Heart disease and stroke statistics–2011 update: a report from the American Heart Association. Circulation.

[CR72] Rosamond WD, Chambless LE, Heiss G, Mosley TH, Coresh J, Whitsel E, Wagenknecht L, Ni H, Folsom AR (2012). Twenty-two-year trends in incidence of myocardial infarction, coronary heart disease mortality, and case fatality in 4 US communities, 1987–2008. Circulation.

[CR73] Rose RA, Jiang H, Wang X, Helke S, Tsoporis JN, Gong N, Keating SC, Parker TG, Backx PH, Keating A (2008). Bone marrow-derived mesenchymal stromal cells express cardiac-specific markers, retain the stromal phenotype, and do not become functional cardiomyocytes in vitro. Stem Cells.

[CR74] Ruger BM, Breuss J, Hollemann D, Yanagida G, Fischer MB, Mosberger I, Chott A, Lang I, Davis PF, Hocker P, Dettke M (2008). Vascular morphogenesis by adult bone marrow progenitor cells in three-dimensional fibrin matrices. Differentiation.

[CR75] Schuleri KH, Feigenbaum GS, Centola M, Weiss ES, Zimmet JM, Turney J, Kellner J, Zviman MM, Hatzistergos KE, Detrick B, Conte JV, McNiece I, Steenbergen C, Lardo AC, Hare JM (2009). Autologous mesenchymal stem cells produce reverse remodelling in chronic ischaemic cardiomyopathy. Eur Heart J.

[CR76] Schwartz SD, Hubschman JP, Heilwell G, Franco-Cardenas V, Pan CK, Ostrick RM, Mickunas E, Gay R, Klimanskaya I, Lanza R (2012). Embryonic stem cell trials for macular degeneration: a preliminary report. Lancet.

[CR77] Shake JG, Gruber PJ, Baumgartner WA, Senechal G, Meyers J, Redmond JM, Pittenger MF, Martin BJ (2002). Mesenchymal stem cell implantation in a swine myocardial infarct model: engraftment and functional effects. Ann Thorac Surg.

[CR78] Sheng CC, Zhou L, Hao J (2013). Current stem cell delivery methods for myocardial repair. Biomed Res Int.

[CR79] Smart N, Risebro CA, Melville AA, Moses K, Schwartz RJ, Chien KR, Riley PR (2007). Thymosin beta4 induces adult epicardial progenitor mobilization and neovascularization. Nature.

[CR80] Smith RR, Barile L, Cho HC, Leppo MK, Hare JM, Messina E, Giacomello A, Abraham MR, Marban E (2007). Regenerative potential of cardiosphere-derived cells expanded from percutaneous endomyocardial biopsy specimens. Circulation.

[CR81] Strauer BE, Brehm M, Zeus T, Gattermann N, Hernandez A, Sorg RV, Kogler G, Wernet P (2001). [Intracoronary, human autologous stem cell transplantation for myocardial regeneration following myocardial infarction]. Dtsch Med Wochenschr.

[CR82] Suncion VY, Ghersin E, Fishman JE, Zambrano JP, Karantalis V, Mandel N, Nelson KH, Gerstenblith G, DiFede Velazquez DL, Breton E, Sitammagari K, Schulman IH, Taldone SN, Williams AR, Sanina C, Johnston PV, Brinker J, Altman P, Mushtaq M, Trachtenberg B, Mendizabal AM, Tracy M, Da Silva J, McNiece IK, Lardo AC, George RT, Hare JM, Heldman AW (2014). Does transendocardial injection of mesenchymal stem cells improve myocardial function locally or globally?: an analysis from the percutaneous stem cell injection delivery effects on neomyogenesis (POSEIDON) randomized trial. Circ Res.

[CR83] Takahashi K, Yamanaka S (2006). Induction of pluripotent stem cells from mouse embryonic and adult fibroblast cultures by defined factors. Cell.

[CR84] Takahashi K, Tanabe K, Ohnuki M, Narita M, Ichisaka T, Tomoda K, Yamanaka S (2007). Induction of pluripotent stem cells from adult human fibroblasts by defined factors. Cell.

[CR85] Tateishi K, Ashihara E, Takehara N, Nomura T, Honsho S, Nakagami T, Morikawa S, Takahashi T, Ueyama T, Matsubara H, Oh H (2007). Clonally amplified cardiac stem cells are regulated by Sca-1 signaling for efficient cardiovascular regeneration. J Cell Sci.

[CR86] Taylor DA, Atkins BZ, Hungspreugs P, Jones TR, Reedy MC, Hutcheson KA, Glower DD, Kraus WE (1998). Regenerating functional myocardium: improved performance after skeletal myoblast transplantation. Nat Med.

[CR87] The Lancet Editors (2014). Expression of concern: the SCIPIO trial. Lancet.

[CR88] Unno K, Jain M, Liao R (2012). Cardiac side population cells: moving toward the center stage in cardiac regeneration. Circ Res.

[CR89] Urbanek K, Rota M, Cascapera S, Bearzi C, Nascimbene A, De Angelis A, Hosoda T, Chimenti S, Baker M, Limana F, Nurzynska D, Torella D, Rotatori F, Rastaldo R, Musso E, Quaini F, Leri A, Kajstura J, Anversa P (2005). Cardiac stem cells possess growth factor-receptor systems that after activation regenerate the infarcted myocardium, improving ventricular function and long-term survival. Circ Res.

[CR90] Valina C, Pinkernell K, Song YH, Bai X, Sadat S, Campeau RJ, Le Jemtel TH, Alt E (2007). Intracoronary administration of autologous adipose tissue-derived stem cells improves left ventricular function, perfusion, and remodelling after acute myocardial infarction. Eur Heart J.

[CR91] van Berlo JH, Kanisicak O, Maillet M, Vagnozzi RJ, Karch J, Lin S-CJ, Middleton RC, Marban E, Molkentin JD (2014). c-kit + cells minimally contribute cardiomyocytes to the heart. Nature.

[CR92] van Tuyn J, Atsma DE, Winter EM, van der Velde-van DI, Pijnappels DA, Bax NA, Knaan-Shanzer S, Gittenberger-de Groot AC, Poelmann RE, van der Laarse A, van der Wall EE, Schalij MJ, de Vries AA (2007). Epicardial cells of human adults can undergo an epithelial-to-mesenchymal transition and obtain characteristics of smooth muscle cells in vitro. Stem Cells.

[CR93] Veltman CE, Soliman OI, Geleijnse ML, Vletter WB, Smits PC, ten Cate FJ, Jordaens LJ, Balk AH, Serruys PW, Boersma E, van Domburg RT, van der Giessen WJ (2008). Four-year follow-up of treatment with intramyocardial skeletal myoblasts injection in patients with ischaemic cardiomyopathy. Eur Heart J.

[CR94] Vrtovec B, Poglajen G, Lezaic L, Sever M, Socan A, Domanovic D, Cernelc P, Torre-Amione G, Haddad F, Wu JC (2013). Comparison of transendocardial and intracoronary CD34+ cell transplantation in patients with nonischemic dilated cardiomyopathy. Circulation.

[CR95] Wakitani S, Saito T, Caplan AI (1995). Myogenic cells derived from rat bone marrow mesenchymal stem cells exposed to 5-azacytidine. Muscle Nerve.

[CR96] Wang X, Hu Q, Nakamura Y, Lee J, Zhang G, From AH, Zhang J (2006). The role of the sca-1+/CD31- cardiac progenitor cell population in postinfarction left ventricular remodeling. Stem Cells.

[CR97] Wessels A, Perez-Pomares JM (2004). The epicardium and epicardially derived cells (EPDCs) as cardiac stem cells. Anat Rec A: Discov Mol Cell Evol Biol.

[CR98] White HD, Aylward PE, Huang Z, Dalby AJ, Weaver WD, Barvik S, Marin-Neto JA, Murin J, Nordlander RO, van Gilst WH, Zannad F, McMurray JJ, Califf RM, Pfeffer MA (2005). Mortality and morbidity remain high despite captopril and/or Valsartan therapy in elderly patients with left ventricular systolic dysfunction, heart failure, or both after acute myocardial infarction: results from the Valsartan in Acute Myocardial Infarction Trial (VALIANT). Circulation.

[CR99] Williams AR, Trachtenberg B, Velazquez DL, McNiece I, Altman P, Rouy D, Mendizabal AM, Pattany PM, Lopera GA, Fishman J, Zambrano JP, Heldman AW, Hare JM (2011). Intramyocardial stem cell injection in patients with ischemic cardiomyopathy: functional recovery and reverse remodeling. Circ Res.

[CR100] Williams AR, Hatzistergos KE, Addicott B, McCall F, Carvalho D, Suncion V, Morales AR, Da Silva J, Sussman MA, Heldman AW, Hare JM (2013). Enhanced effect of combining human cardiac stem cells and bone marrow mesenchymal stem cells to reduce infarct size and to restore cardiac function after myocardial infarction. Circulation.

[CR101] Yin AH, Miraglia S, Zanjani ED, Almeida-Porada G, Ogawa M, Leary AG, Olweus J, Kearney J, Buck DW (1997). AC133, a novel marker for human hematopoietic stem and progenitor cells. Blood.

[CR102] Zaruba MM, Soonpaa M, Reuter S, Field LJ (2010). Cardiomyogenic potential of C-kit(+)-expressing cells derived from neonatal and adult mouse hearts. Circulation.

[CR103] Zhao T, Zhang ZN, Rong Z, Xu Y (2011). Immunogenicity of induced pluripotent stem cells. Nature.

[CR104] Zhou B, Honor LB, He H, Ma Q, Oh JH, Butterfield C, Lin RZ, Melero-Martin JM, Dolmatova E, Duffy HS, Gise A, Zhou P, Hu YW, Wang G, Zhang B, Wang L, Hall JL, Moses MA, McGowan FX, Pu WT (2011). Adult mouse epicardium modulates myocardial injury by secreting paracrine factors. J Clin Invest.

